# Trimethoprim-sulfamethoxazole and the risk of early severe infection in elderly-onset myeloperoxidase-antineutrophil cytoplasmic antibody-associated vasculitis

**DOI:** 10.1186/s12882-025-04695-y

**Published:** 2025-12-17

**Authors:** Shun Yoshida, Kohei Yamamura, Keiichi Osano, Miho Shikata, Toshihisa Ishii, Makiko Konishi, Kazuya Takahashi, Daiki Nakagomi, Kohei Uchimura, Ayumu Nakashima

**Affiliations:** 1 Department of Nephrology, Graduate School of Medicine, University of Yamanashi, 1110 Shimokato Chuo, Yamanashi, 409–3898 Japan; 2 Department of Rheumatology, Graduate School of Medicine, University of Yamanashi, 1110 Shimokato Chuo, Yamanashi, 409–3898 Japan

**Keywords:** MPO-ANCA-associated vasculitis, ANCA-associated glomerulonephritis, Elderly, MPA

## Abstract

**Background:**

Antineutrophil cytoplasmic antibody (ANCA)-associated vasculitis (AAV) has improved survival outcomes with advancements in immunosuppressive therapies; however, the increased incidence of infections remains a significant concern. Reports on infection risk factors other than age are limited, especially in older adults with AAV who are at a higher risk.

**Methods:**

We aimed to identify the risk factors for early-phase infection during treatment initiation in older adults (aged ≥ 75 years) with myeloperoxidase (MPO)-ANCA-positive AAV (MPO-AAV). This was a single-center, retrospective observational study that included 50 patients who were classified as having microscopic polyangiitis. Severe infections were defined as those requiring hospitalization within six months of treatment initiation.

**Results:**

Severe infections occurred in 17 (34%) patients. No statistically significant associations were observed between disease incidence and either combined immunosuppressive therapy or organ-specific lesions. However, trimethoprim-sulfamethoxazole (TMP-SMX) was significantly associated with a reduced risk of infection. This association remained statistically significant after adjustment for sex (hazard ratio, 0.23; 95% CI, 0.08–0.64; *P* < 0.01).

**Conclusions:**

In older adults with MPO-AAV, TMP-SMX use was associated with a reduced risk of early severe infection. Although limited by the observational nature of the study, these findings suggest a potential role for TMP-SMX prophylaxis in high-risk elderly populations.

**Clinical trial number:**

Not applicable.

**Supplementary Information:**

The online version contains supplementary material available at 10.1186/s12882-025-04695-y.

## Background

Antineutrophil cytoplasmic antibody (ANCA)-associated vasculitis (AAV) is a form of small-vessel vasculitis that affects multiple organs [1]. Microscopic polyangiitis (MPA) accounts for most cases of AAV in Southern Europe, China, and Japan [2, 3]. Most patients with MPA are positive for myeloperoxidase (MPO)-ANCA and have a median age of 71 years at onset [2]. This is somewhat older than proteinase-3 (PR3)-ANCA-positive granulomatosis with polyangiitis (GPA), which is more common in Northern Europe, Australia, and the United States [2–6].

Remission induction therapy often involves a combination of immunosuppressive agents, such as rituximab (RTX) or cyclophosphamide (CY), and corticosteroids [7, 8]. Infections are one of the most severe adverse events associated with these treatments [9, 10].

Severe infections frequently occur in the early phase (within six months) after remission induction therapy [11]. Numerous studies have highlighted a strong association between advanced age and risk of infections [7, 10, 12–14]. Although infection-related complications following treatment initiation are associated with increased mortality, the absence of treatment is associated with an elevated mortality risk [15, 16]. Therefore, in older adults with AAV, meticulous attention should be paid to preventing infections during therapy initiation, with careful monitoring throughout the subsequent clinical course.

In addition to advanced age, various other risk factors for infection following AAV treatment have been reported. These include smoking status, Birmingham Vasculitis Activity Score (BVAS), renal impairment, pulmonary lesions, leukopenia, CY or RTX regimens, and initial dose of prednisolone (PSL) [10, 11, 13–15, 17–25]. However, studies focusing specifically on infection risk factors in older adults with AAV are limited [11, 16, 23]. For example, treatment-related factors, such as RTX and high-dose PSL, have been identified as risk factors for infection; however, these findings often involve cohorts with a high proportion of PR3-ANCA-positive patients. Similarly, Japanese studies predominantly focusing on MPA have reported that the initial PSL dose is an infection risk factor [23].

In line with these findings, current guidelines advocate reducing PSL doses when corticosteroids are combined with immunosuppressive agents [7, 8]. Nevertheless, the risk factors for infection in older adults with myeloperoxidase-antineutrophil cytoplasmic antibody-positive AAV (MPO-AAV) receiving guideline-concordant therapy remain unclear. This study aimed to identify the factors associated with early infection following remission induction therapy in older adults with MPO-AAV.

## Methods

This was a single-center, retrospective, observational study. Among 145 patients with MPO-AAV who were newly diagnosed and underwent remission-induction therapy between April 2012 and March 2024, those aged ≥ 75 years with available follow-up data at six months post-therapy were included (Additional file 1). The six-month follow-up period after treatment was established based on previous reports indicating that this is the most clinically significant period during which infections occur most frequently after remission-induction therapy [11, 17, 26]. All patients were classified as having MPA according to the 2022 American College of Rheumatology/European Alliance of Associations for Rheumatology vasculitis classification criteria [27–29]. Patient data, including demographic information at admission, laboratory parameters at treatment initiation, administered therapies, time to infection onset post-treatment, infection types, and outcomes, such as mortality or relapse, were collected from medical records. As part of the baseline patient background, medical records were used to assess infection history, comorbidities, and physical function. History of infection was defined as any infection requiring hospitalization or outpatient treatment within 12 months of AAV diagnosis. Comorbidities were scored using the Charlson Comorbidity Index [30]. Activities of Daily Living (ADL) were evaluated using the Barthel Index (BI), with a BI score of < 100 indicating ADL dependence (dependent) [31]. Remission-induction therapy was categorized into four groups: corticosteroids alone, RTX monotherapy, CY monotherapy, and combination therapy with RTX and CY. Dialysis dependence was defined as the requirement for dialysis therapy at the time of hospital admission or initiation of treatment.

Data for this retrospective observational study were accessed from December 1, 2024, to February 29, 2025. Although, the authors had the potential to access information that could identify individual participants (e.g., patient IDs for data linkage), no identifiable information was accessed, recorded, or used for analysis during or after data collection. All data used in this study were de-identified to protect patient privacy. The patients were categorized into an “infection group” (those who developed severe infections within six months) and a “non-infection group” (those without infections). The patient characteristics and treatments were compared between the two groups. Severe infections were defined as bacterial infections or pneumocystis pneumonia (PCP) that required inpatient treatment. Patients with viral infections were excluded.

## Statistical analysis

Data are presented as mean values ± standard deviation or median and interquartile range (25–75th percentiles). The Mann–Whitney U or χ2 test was used to compare the groups. Additionally, we estimated infection rate using Kaplan–Meier analysis. The difference in infection rates between the two groups was examined using a log-rank χ² test. Univariate and multivariate Cox regression analyses of infection-free survival were presented as hazard ratios (HR) and 95% confidence intervals (CI). All analyses were performed using JMP Pro 18 software (SAS Institute Inc., Cary, NC, USA).

## Results

The mean age of the 50 patients in the study was 80.1 ± 4.0 years, and 27 (54%) were male. Seventeen (34%) patients were classified into the infection group, and 33 were assigned to the non-infection group. The onset of infections occurred evenly over the six months following treatment initiation (Additional file 2). Respiratory infections were the most common type, accounting for 41.2% (*n* = 7) of cases (Additional file 3).

The baseline characteristics of the patients in each group are shown in Table 1. The proportion of males was higher in the infection group than in the non-infection group (76.5% vs. 42.4%, *P* = 0.04), In contrast, there were no significant differences in age, diabetes, or smoking history between the groups. Regarding laboratory findings, the infection group exhibited higher proteinuria levels at diagnosis (1.68 g/gCr vs. 0.55 g/gCr, *P* = 0.03), whereas serum creatinine, estimated glomerular filtration rate (eGFR), and MPO-ANCA titers were comparable between the groups. Regarding treatment-related factors, no significant differences were observed in the initial corticosteroid doses, methyl PSL pulse, CY use, or RTX use between the two groups. However, the use of trimethoprim-sulfamethoxazole (TMP-SMX) was notably lower in the infected group (35.3% vs. 84.8%, *P* < 0.01).

**Table 1:** Patient characteristics at diagnosis

	All patients (*n* = 50)	Infection group (*n* = 17)	Non-infection group (*n* = 33)	*P*-value
Age, years	80.1 ± 4.0	81.2 ± 3.7	80.8 ± 4.1	0.70
Male, n (%)	27 (54.0)	13 (76.5)	14 (42.4)	0.04
Hypertension, n (%)	30 (60.0)	13 (76.5)	17 (51.5)	0.13
Diabetes, n (%)	9 (18.0)	3 (17.6)	6 (18.2)	1.00
Prior infection history, n (%)	1 (2.0)	0 (0)	1 (3.0)	1.00
Charlson Comorbidity index	5 [4–6]	5 [5–6.5]	5 [4–6]	0.75
BI < 100 (Dependent), n (%)	15 (30.0)	7 (41.2)	8 (24.2)	0.33
Smoking status, n (%)	27 (54.0)	11 (64.7)	16 (48.5)	0.37
Duration diagnosis (months)	2 [1–5]	2 [1–4]	2.5 [1–5]	0.55
MPO-ANCA, n (%)	50 (100)	17 (100)	33 (100)	
ANCA titer (U/mL)	127 [48.6–463.8]	238 [65.5–430]	114 [37.6–557.5]	0.47
creatinine (mg/dL)	2.49 ± 3.03	2.25 ± 1.87	2.61 ± 3.50	0.32
eGFR (mL/min/1.73)	40.96 ± 27.64	35.00 ± 23.19	44.03 ± 29.54	0.36
UPCR (g/gCr)	0.87 [0.31–2.43]	1.68 [0.82–3.16]	0.55 [0.23–2.01]	0.03
KL-6 (U/mL)	235 [159–371]	199 [117–354]	270 [180–372]	0.25
CRP (mg/dL)	5.7 [2.0–10.7]	10.4 [2.9–12.9]	5.4 [1.5–9.9]	0.19
IgG (mg/dL)	1754 ± 521	1741 ± 449	1759 ± 560	1.00
CSs (initial dose, mg/day)	30 [25–40]	30 [20–42.5]	30 [30–40]	0.98
CSs only, n (%)	16 (32.0)	6 (35.3)	10 (30.3)	0.76
mPSL pulse, n (%)	10 (20.0)	5 (29.4)	5 (15.2)	0.28
CY use, n (%)	12 (24.0)	4 (23.5)	8 (24.2)	1.00
RTX use, n (%)	24 (48.0)	8 (47.1)	16 (48.5)	1.00
CY + RTX, n (%)	2 (4.0)	1 (5.9)	1 (3.0)	1.00
PE, n (%)	5 (10.0)	2 (11.8)	3 (9.1)	1.00
TMP-SMX use, n (%)	34 (68.0)	6 (35.3)	28 (84.8)	< 0.01

Values are presented as mean ± standard deviation or median [interquartile range] for continuous variables, and number (%) for categorical variables. P-values from the Mann-Whitney U test or chi-squared test are shown for comparisons between the infection and non-infection groups. Abbreviations: MPO-ANCA, myeloperoxidase antineutrophil cytoplasmic antibody; BI, Barthel Index; IgG, immunoglobulin G; eGFR, estimated glomerular filtration rate; UPCR, urinary protein-to-creatinine ratio; KL-6, Krebs von den Lungen-6; CRP, C-reactive protein; CS, corticosteroid; mPSL, methylprednisolone; CY, cyclophosphamide; RTX, rituximab; PE, plasma exchange; TMP-SMX, trimethoprim-sulfamethoxazole

Details of the organ involvement associated with AAV are presented in Table 2. No significant differences were observed between the two groups in BVAS scores or in the prevalence of organ-specific lesions.

**Table 2:** BVAS and organ involvement at diagnosis

	All patients (*n* = 50)	Infection group (*n* = 17)	Non-infection group (*n* = 33)	*P*-value
BVAS, points	15 [12–18]	14 [13–18]	15 [12–19]	0.81
General, n (%)	33 (66.0)	10 (58.8)	23 (69.7)	0.53
Skin, n (%)	4 (8.0)	3 (17.6)	1 (3.0)	0.11
Joint, n (%)	4 (8.0)	0 (0.0)	4 (12.1)	0.29
Mucous membranes, n (%)	2 (4.0)	0 (0.0)	2 (6.1)	0.54
Eyes, n (%)	5 (10.0)	1 (5.9)	4 (12.1)	0.65
ENT, n (%)	9 (18.0)	4 (23.5)	5 (15.2)	0.47
Lung, n (%)	30 (60.0)	13 (76.5)	17 (51.5)	0.13
IP, n (%)	27 (54.0)	12 (70.6)	15 (45.5)	0.14
Alveolar hemorrhage, n (%)	3 (6.0)	1 (5.9)	2 (6.1)	1.00
Cardiovascular, n (%)	4 (8.0)	2 (11.8)	2 (6.1)	0.60
Gastrointestinal, n (%)	0 (0)	0 (0)	0 (0)	-
Kidney, n (%)	45 (90.0)	17 (100)	28 (84.8)	0.15
Renal limited, n (%)	3 (6.0)	1 (5.9)	2 (6.1)	1.00
RPGN, n (%)	23 (46.0)	10 (58.8)	13 (39.4)	0.24
Dialysis dependence, n (%)	5 (10.0)	2 (11.8)	3 (9.1)	1.00
CNS*, n (%)	8 (16.0)	2 (11.8)	6 (18.2)	0.70
Neuropathy, n (%)	12 (24.0)	3 (17.6)	9 (27.3)	0.51

Values are presented as median [interquartile range] for the BVAS, and number (%) for organ involvement. P-values from the Mann-Whitney U test or chi-squared test are shown for comparisons between the infection and non-infection groups. Abbreviations: BVAS, Birmingham Vasculitis Activity Score; CNS, central nervous system; ENT, ear, nose, and throat; IP, interstitial pneumonia; RPGN, rapidly progressive glomerulonephritis. *CNS involvement included headache (two patients), cerebrovascular accident (five patients), and cognitive dysfunction (one patient)

Major events occurring within six months of treatment initiation are summarized in Table 3. There were four (8.0%) mortalities in total, all of which were attributed to infections. End-stage renal disease at six months was observed in eight (16%) patients, and vasculitis relapse occurred in five patients (10%). No significant differences in the incidence of these major events were observed between the two groups.

**Table 3:** Major events occurring within six months of treatment initiation

	All patients (*n* = 50)	Infection group (*n* = 17)	Non-infection group (*n* = 33)	*P*-value
ESRD, n (%)	8 (16.0)	3 (17.6)	5 (15.2)	1.00
Death, n (%)	4 (8.0)	4 (23.5)	0 (0)	0.01
Relapse, n (%)	5 (10.0)	3 (17.6)	2 (6.1)	0.32

Values are presented as number (%). P-values from the chi-squared test or Fisher’s exact test are shown for comparisons between the infection and non-infection groups. Abbreviations: ESRD, end-stage renal disease. ESRD status was assessed at six months

To identify factors associated with the onset of the first infection within six months of treatment initiation, we calculated HRs using a Cox proportional hazards model, as shown in Table 4. In the univariate analysis, TMP-SMX use was significantly associated with infection risk reduction (hazard ratio [HR], 0.20; 95% CI: 0.07–0.55, *P* < 0.01). Sex also showed a statistically significant association with increased infection risk (HR, 3.08; 95% CI: 1.00-9.47, *P* = 0.05). After adjusting for sex in the multivariate analysis, TMP-SMX use remained significantly associated with a reduced risk of infection (HR, 0.23; 95% CI: 0.08–0.64, *P* < 0.01). However, the association between sex and the risk of infection was no longer significant in the adjusted model. Other variables, including age, BI < 100 (dependent), presence of interstitial pneumonia, eGFR, proteinuria levels, presence of rapidly progressive glomerulonephritis, initial CS dose, RTX use and BVAS, showed no significant associations.

**Table 4:** Univariate and multivariate cox proportional hazards regression analysis for infection-free survival

Variables	Univariate analysis		Multivariate analysis	
	Hazard ratio (95% CI)	*P*-value	Hazard ratio (95% CI)	*P*-value
Age, years	1.02 (0.90–1.15)	0.72		
Male	3.08 (1.00–9.47)	0.05	2.42 (0.78–7.55)	0.13
BI < 100 (dependent)	2.09 (0.79–5.52)	0.14		
IP	2.30 (0.81–6.55)	0.12		
eGFR, mL/min/1.73 m^2^	0.99 (0.97–1.01)	0.26		
UPCR, g/gCr	1.03 (0.82–1.18)	0.76		
RPGN	1.98 (0.75–5.2)	0.17		
Initial CS dose, mg	1.01 (0.96–1.05)	0.77		
CS only (vs. RTX or CY use)	1.20 (0.45–3.26)	0.71		
RTX use	1.01 (0.39–2.63)	0.98		
TMP-SMX use	0.20 (0.07–0.55)	< 0.01	0.23 (0.08–0.64)	< 0.01
BVAS, points	1.04 (0.95–1.13)	0.40		

Hazard ratios (HR) and 95% confidence intervals (CI) were presented. In the multivariate analysis, variables with a p-value less than 0.1 in the univariate analysis were selected as candidates for inclusion. A stepwise forward selection method was applied, which resulted in a final model that included sex and TMP-SMX use. Abbreviations: BI, Barthel index; IP, interstitial pneumonia; eGFR, estimated glomerular filtration rate; UPCR, urinary protein-to-creatinine ratio; RPGN, rapidly progressive glomerulonephritis; CS, corticosteroid; RTX, rituximab; CY, cyclophosphamide; TMP-SMX, Trimethoprim-sulfamethoxazole; BVAS, Birmingham Vasculitis Activity Score; CI, confidence interval

**Fig. 1:**
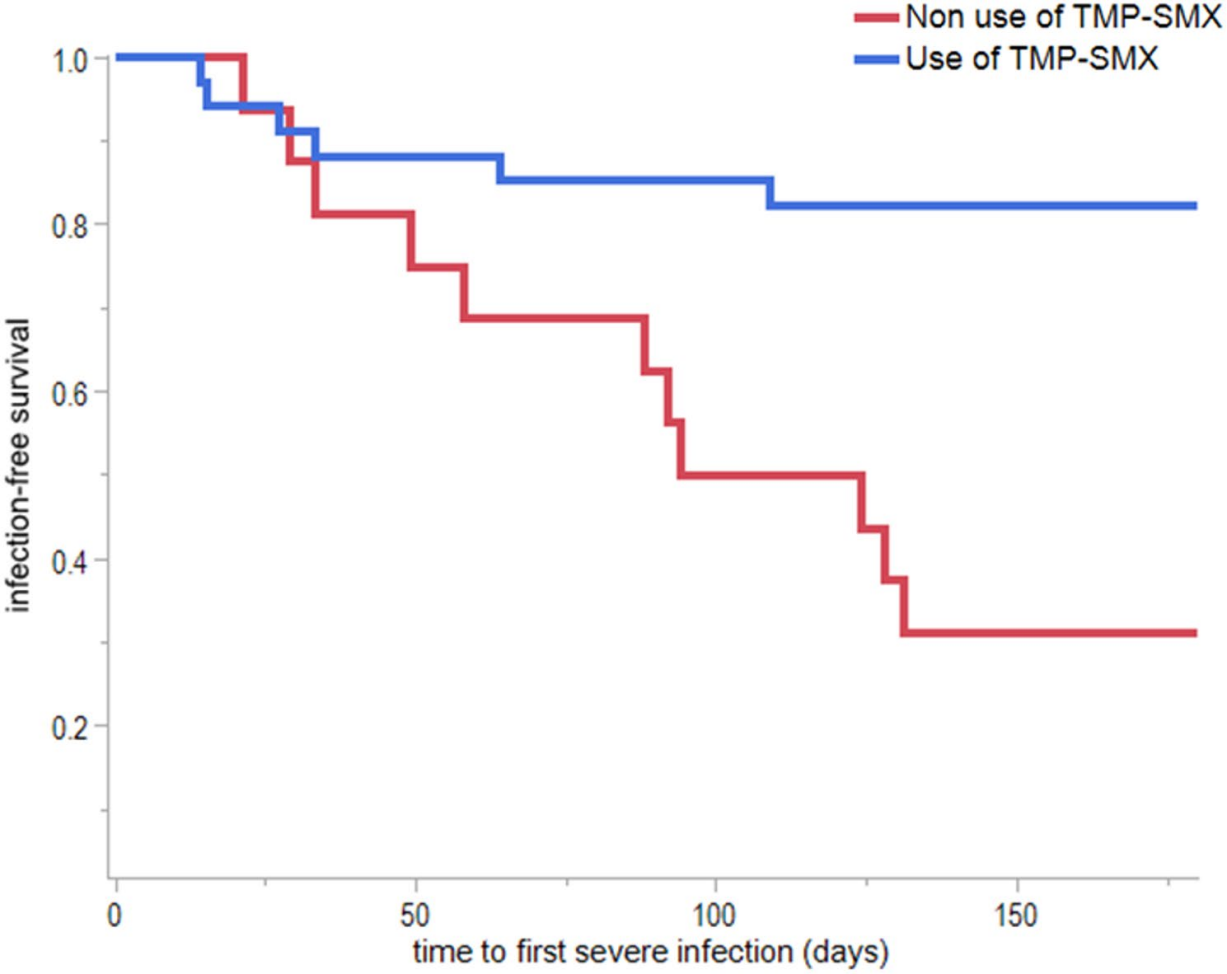
Kaplan–Meier curves showing infection-free survival stratified by trimethoprim-sulfamethoxazole (TMP-SMX) use. Patients receiving TMP-SMX had a significantly higher infection-free survival rate than those not receiving TMP-SMX (log-rank χ² test, *P* < 0.01)

Figure 1 shows the Kaplan–Meier curve for infection-free survival stratified by TMP-SMX use. A significant difference was observed between the two groups, with patients receiving TMP-SMX exhibiting a higher infection-free survival rate than those not receiving TMP-SMX (log-rank χ2 test, *P* < 0.01).

## Discussion

This study aimed to explore risk factors for early infection after therapy initiation in older adults with MPO-AAV. The absence of TMP-SMX was significantly associated with an increased risk of infection. In addition, this association remained statistically significant after adjustment for sex. These findings suggest that TMP-SMX use in older adults with MPO-AAV may reduce the incidence of severe infections within six months of remission induction therapy. TMP-SMX is a prophylactic agent commonly used to prevent PCP and is recommended in the guidelines for patients with AAV receiving RTX, CY, or high-dose corticosteroids [7, 8]. The results of this study indicate that TMP-SMX use in older adults with MPO-AAV may not only prevent PCP but also help suppress the overall risk of infections, regardless of the type of induction therapy administered.

Kronbichler et al. reported that among 192 patients with AAV treated with RTX, 25.5% developed severe infections, and the use of TMP-SMX was associated with a reduced risk of severe infections (HR, 0.30; 95% CI: 0.13–0.69) [21]. Additionally, a post-hoc analysis of the RAVE trial, a large-scale randomized controlled trial (RCT) revealed that 18 of 197 (9.1%) patients developed early infections [26]. Regardless of whether RTX or CY was used, patients who received TMP-SMX had a lower infection rate. These two reports differ from the present study in that their cohorts were younger and included a majority of patients with GPA, whereas the current study focused on older adults with MPA.

Reports on the efficacy of TMP-SMX in specifically targeting MPA are limited. However, a study involving patients with AAV, nearly 70% of whom had MPA, demonstrated a reduction in infection risk with TMP-SMX [32]. Notably, the infection rate in that study was 18%, and the cohort included relatively few high-risk patients, such as those treated with RTX or CY, which are associated with a higher infection risk.

In this study, respiratory tract infections were the most frequently observed severe infections. This result is consistent with the findings of a prior survey by Kronbichler et al. [21]. Although a significant association between pulmonary complications and the risk of respiratory infections has been demonstrated, no such association was observed in our study. This discrepancy may be due to the limited sample size of our cohort.

The most significant feature of this study is its exclusive focus on MPO-AAV (100% MPA) and its target population of extremely elderly individuals aged ≥ 75 years. This patient group reflects real-world clinical practice in East Asia, characterized by an exceptionally high infection risk, in which treatment choices directly impact prognosis. In previous RCTs and large-scale reports, the majority of cases were GPA and included younger patients [33, 34]. Therefore, studies focusing on specific populations of elderly onset MPO-AAV have been limited. Fundamental differences in patient backgrounds, such as age, ANCA type, and regional characteristics, do not merely reflect cohort diversity but also result in significant differences in the quality of comorbidities, the characteristics of immune responses, and even the risk of developing infections, all of which fundamentally impact clinical outcomes. This study was designed to complement existing evidence. Compared with previous studies, the infection rate in this cohort was notably high (34%). Despite this high-risk patient profile, our findings demonstrate the efficacy of TMP-SMX in reducing infection incidence, underscoring its clinical importance.

This study has some limitations. First, this was a small-scale, single-center, retrospective observational study. Although we attempted to control for many critical confounding factors, we cannot rule out the possibility that unmeasured or unknown confounders may have influenced the results. We focused on older adults with relatively preserved ADL who attended the outpatient department. Additionally, the analysis was limited to infections occurring within six months of treatment initiation, and infections during the subsequent maintenance therapy period were not included. Therefore, to confirm our findings in an elderly population over a more extended period and to establish the safest and most effective strategy, pragmatic trials comparing the optimal duration (e.g., 6 vs. 12 months) or dosage of TMP-SMX prophylaxis are desirable.

In conclusion, among the elderly patients with MPO-AAV, TMP-SMX use was associated with the occurrence of early severe infections after remission-induction therapy. Therefore, in older adults at high risk of infection, TMP-SMX should be actively prescribed not only for PCP prevention but also as a broader measure to mitigate infection risk.

## Supplementary Information

Below is the link to the electronic supplementary material.

Supplementary Material 1

## Data Availability

All data generated or analyzed during this study are included in this published article and its supplementary information files.

## Abbreviations

AAV: ANCA-associated vasculitis
ADL: Activities of Daily Living
ANCA: Antineutrophil cytoplasmic antibody
BI: Barthel Index
BVAS: Birmingham Vasculitis Activity Score
CI: Confidence intervals
CY: Cyclophosphamide
eGFR: estimated glomerular filtration rate
GPA: Granulomatosis with polyangiitis
HR: Hazard ratios
MPA: Microscopic polyangiitis
MPO: Myeloperoxidase
MPO-AAV: Myeloperoxidase-antineutrophil cytoplasmic antibody-positive AAV
PCP: Pneumocystis pneumonia
PR3: Proteinase-3
PSL: Prednisolone
RCT: randomized controlled trial
RTX: Rituximab
TMP-SMX: Trimethoprim-sulfamethoxazole
