# Supplementary material for: Trimethoprim-sulfamethoxazole and the risk of early severe infection in elderly-onset myeloperoxidase-antineutrophil cytoplasmic antibody-associated vasculitis
Source: BMC Nephrol. 2025 Dec 17;27:58. doi: 10.1186/s12882-025-04695-y (PMC12821867; doi:10.1186/s12882-025-04695-y)
Supplement: Supplementary file 1 — Supplementary Material 1 [file 12882_2025_4695_MOESM1_ESM.docx]

**Research article**

**Trimethoprim-sulfamethoxazole and the risk of early severe infection in elderly-onset myeloperoxidase-antineutrophil cytoplasmic antibody-associated vasculitis**

Shun Yoshida ^1*^, Kohei Yamamura ^1^, Keiichi Osano ^1^, Miho Shikata ^1^, Toshihisa Ishii ^1^, Makiko Konishi ^1^, Kazuya Takahashi ^1^, Daiki Nakagomi ^2^, Kohei Uchimura ^1^ and Ayumu Nakashima ^1*^

^1^Department of Nephrology, Graduate School of Medicine, University of Yamanashi, 1110 Shimokato Chuo, Yamanashi 409-3898, Japan

^2^Department of Rheumatology, Graduate School of Medicine, University of Yamanashi, 1110 Shimokato Chuo, Yamanashi 409-3898, Japan

***Corresponding Author:**

Shun Yoshida, MD

Department of Nephrology, Graduate School of Medicine, University of Yamanashi

1110 Shimokato Chuo, Yamanashi 409-3898, Japan

TEL: +81-55-273-2288; FAX: +81-55-273-2280

Email: yoshidas@yamanashi.ac.jp

Ayumu Nakashima, MD, PhD., FACP, FASN

Department of Nephrology, Graduate School of Medicine, University of Yamanashi

1110 Shimokato Chuo, Yamanashi 409-3898, Japan

TEL: +81-55-273-2288; FAX: +81-55-273-2280

Email: [a.nakashima@yamanashi.ac.jp](mailto:a.nakashima@yamanashi.ac.jp)

# **Additional Files**

AAV patients (n = 145)

Excluded:

- Age < 75 years (n = 48)
- PR3-ANCA positive or ANCA negative (n=17)
- No treatment (n=3)

Age ≥ 75 years (n = 77)

Excluded:

- Acute phase death (n=5)
- No follow-up within 6 months (n = 22)

**Final analyzed cases: n = 50**

**Additional file 1. Flowchart of patient selection for analysis in an elderly myeloperoxidase-antineutrophil cytoplasmic antibody-associated vasculitis (MPO-AAV) patient cohort.**

To extract elderly MPO-AAV cases, patients with proteinase 3-antineutrophil cytoplasmic antibody (PR3-ANCA) positivity, ANCA (antineutrophil cytoplasmic antibody) negativity, no treatment, and those under 75 years of age were excluded. Furthermore, cases lacking a six-month follow-up period or those with acute phase death (death during induction therapy) were also excluded, resulting in a final cohort of 50 patients for this study's analysis.


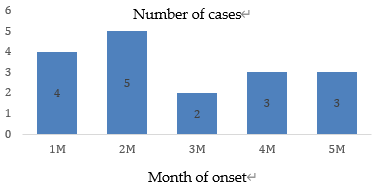


**Additional file 2. Timing of Infections Post-Treatment**

The onset of infections following treatment occurred evenly throughout the first six months. No specific temporal clustering of infection cases was observed during this period.

**Additional file 3. Type of infections in patients with severe infections.**

|  | No. of patients with severe infections, n (%) | 17 (34.0) |
| --- | --- | --- |
|  | Pneumonia, n (%) | 7 (41.2) |
|  | Bacteremia | 2 (11.8) |
|  | Urinary tract infection | 2 (11.8) |
|  | Nonspecific infection | 2 (11.8) |
|  | Aspergillus | 1 (5.9) |
|  | Pneumocystis pneumonia | 3 (17.6) |

This table presents the number and percentage of different types of infections observed among patients who developed severe infections.
